# Video-based assessment (VBA) of an open, simulated orthopedic surgical procedure: a pilot study using a single-angle camera to assess surgical skill and decision making

**DOI:** 10.1186/s13018-023-03557-3

**Published:** 2023-02-07

**Authors:** Obaid Al-Hubaishi, Troy Hillier, Megan Gillis, William Oxner, Andrew Trenholm, Glen Richardson, Ross Leighton, Andrew Glennie

**Affiliations:** 1 Department of Surgery, Dalhousie University, Halifax, NS Canada; 2 Faculty of Medicine, Dalhousie University, Halifax, NS Canada

**Keywords:** Video-based assessment, Orthopedic surgery, Medical education, Competency-Based Medical Education

## Abstract

**Background:**

Videos have been used in many settings including medical simulation. Limited information currently exists on video-based assessment in surgical training. Effective assessment tools have substantial impact on the future of training. The objectives of this study were as follows: to evaluate the inter-rater reliability of video-based assessment of orthopedic surgery residents performing open cadaveric simulation procedures and to explore the benefits and limitations of video-based assessment.

**Methods:**

A multi-method technique was used. In the quantitative portion, four residents participated in a Surgical Objective Structured Clinical Examination in 2017 at a quaternary care training center. A single camera bird’s-eye view was used to videotape the procedures. Five orthopedic surgeons evaluated the surgical videos using the Ottawa Surgical Competency Operating Room Evaluation. Interclass correlation coefficient was used to calculate inter-rater reliability. In the qualitative section, semi-structured interviews were used to explore the perceived strengths and limitations of video-based assessment.

**Results and discussion:**

The scores using video-based assessment demonstrated good inter-rater reliability (ICC = 0.832, *p* = 0.014) in assessing open orthopedic procedures on cadavers. Qualitatively, the strengths of video-based assessment in this study are its ability to assess global performance and/or specific skills, ability to reassess missed points during live assessment, and potential use for less common procedures. It also allows for detailed constructive feedback, flexible assessment time, anonymous assessment, multiple assessors and serves as a good coaching tool. The main limitations of video-based assessment are poor audio–video quality, and questionable feasibility for assessing readiness for practice.

**Conclusion:**

Video-based assessment is a potential adjunct to live assessment in orthopedic open procedures with good inter-rater reliability. Improving audio–video quality will enhance the quality of the assessment and improve the effectiveness of using this tool in surgical training.

**Supplementary Information:**

The online version contains supplementary material available at 10.1186/s13018-023-03557-3.

## Introduction

Simulation has been used in various medical specialties and is an essential component of Competency-Based Medical Education (CBME). It has recently become an invaluable training adjunct in surgery due to concerns over patient safety, outcomes, decreased resident working hours, and limited clinical encounters due to the COVID-19 pandemic [1, 2]. Martin et al. [3] used cadaveric simulation to demonstrate that residents’ skills and patients’ clinical outcomes can be enhanced with competency-based training. Moore et al. [4] demonstrated that GoPro cameras can be used as a coaching tool, while illustrating how residents can improve psychomotor surgical skills. Simulations can also provide greater insight compared to traditional forms of assessment, as the performance can be reviewed and debriefed often immediately after the simulated encounter directly with the learner**.**

There are few studies, however, that examine the reliability of using open surgical simulation in high-stakes decisions about advancement through training and readiness for practice [5]. Simulations have been used to improve and assess laparoscopic, endovascular, and endoscopic skills performance but we are not aware of studies that have evaluated the feasibility of using video for open skills assessment [2, 6–8]. Video is fundamental to the performance of surgical arthroscopy and has shown good reliability as an evaluation tool in orthopedics [9, 10]. Although video is commonly used to demonstrate complex open techniques for presentation or teaching purposes, it is seldom used as an evaluation tool. As the technology used in evaluating cadaveric surgical simulations improves—such as video-based assessment (VBA), virtual reality, or augmented reality—developing more reliable methods of assessing performance in surgical trainees is imperative. Video recordings may allow evaluation at a national level for various licensing authorities, since demonstrating evidence of appropriate surgical skills is currently lacking or nonexistent in most board examinations.

Establishing reliable assessment tools that are predictive of future success has been a significant challenge in orthopedics [2, 11]. Most of the shortcomings center on the fidelity of the simulations (i.e., trainees and supervising surgeons alike question the reliability of performing and evaluating tasks within a box trainer). With the increased application of CBME or milestone-based evaluation systems, various reliable methods of assessing competencies are needed [12, 13]. Unfortunately, subjective assessment tools continue to dominate decisions about progress in training, with only modest evidence of reliability [13].

## Aim

To assess the inter-rater reliability of video-based assessment (VBA) in a cadaveric simulated open orthopedic surgical procedure and to explore the perceived qualitative strengths and limitations of VBA as judged by the video evaluators.

### Methods

In a quaternary orthopedic residency training program, a Surgical Objective Clinical Skills Examination (S-OSCE) is performed on an annual basis for all senior residents to assess surgical skills and independent decision making. In this unique assessment tool, residents perform full standardized surgical procedures on fresh cadavers, equipped with standard operating room tools and fluoroscopic imaging before and after interacting with simulated patients. Fracture patterns and other pathologies (tendon tears, fractures, etc.) are simulated, and radiographic imaging is produced to correlate with the injury patterns for residents to interpret. Techniques for fracture simulation have been described previously but often involve an osteotome through the bone through incisions away from the planned surgical field [14]. Faculty members directly observe resident performance, using checklists and global rating scores to evaluate competence.

**Fig. 1:**
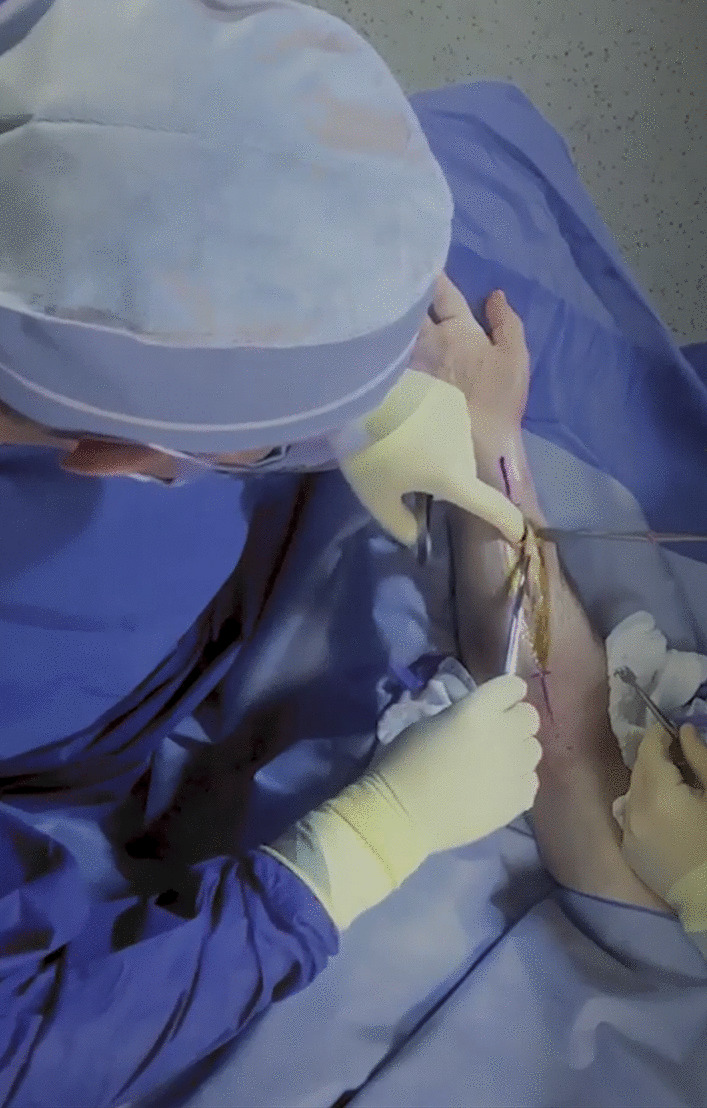
Video snapshot demonstrating the birds-eye view of the surgical site used in the current study

In 2017, four residents (three third-year and one fourth-year) consented to have their S-OSCE videotaped for this pilot study. The recorded videos were of open reduction and internal fixation (ORIF) of a both bone forearm fracture. This is a procedure of medium complexity and an operation defined within the critical competencies through the Royal College of Physicians and Surgeons of Canada (RCPSC). Standard operating room tools, a surgical assist (junior resident), and fluoroscopic imaging were provided. A canon XA40 camcorder was used to audio- and video-record the procedures with a birds-eye view of the surgical field (https://www.canon.ca) (Fig. 1). Purposive sampling was used to select examiners with different scopes of practice and levels of experience in residents’ assessments. Participants were five orthopedic surgeons who consented to participate in VBA and semi-structured interviews to explore the benefits and limitations of VBA. The assessors included members of the training committee of the orthopedics program. The feasibility of VBA for evaluation of an open orthopedic surgical procedure was investigated in this pilot study, with the opportunity for expansion in the future based on examiner feedback.

Residents’ performance of the selected surgical task was assessed using the Ottawa Surgical Competency Operating Room Evaluation (O-SCORE). The O-SCORE has been described in depth and validated previously [15]; briefly, it is a tool developed to measure the competence of surgical residents’ performance of common surgical procedures. The O-SCORE contains eight prompts which are rated on a 5-point scale, two open-ended questions, and one further prompt addressing the readiness for independent practice of the surgical trainee [15].

Following the VBA, O-SCORE results were transferred to a Statistical Package for the Social Sciences (SPSS) (IBM Corp, Armonk, NY, USA) spreadsheet. Inter-rater reliability was calculated using the intraclass correlation coefficient (ICC). The statistical significance of the ICC was determined using the mean O-SCORE rating, absolute-agreement, and a 2-way random-effects model. ICC values less than 0.5 were labeled as poor reliability, values between 0.5 and 0.75 were labeled as moderate reliability, values between 0.75 and 0.9 were labeled as good reliability, and values more than 0.9 were labeled as excellent reliability [16].

For the qualitative analysis, semi-structured 20–40-min interviews with the examiners were conducted by OAH and AG to explore the strengths and limitations of video-based assessment. All interviews were completed face to face or by phone call between March 2021 and May 2021. The collection of identifying information about the examiners was avoided to encourage open responses. The examiners were encouraged to speak freely about their experiences doing video assessments. Interviews were audio-recorded and transcribed, and the transcripts de-identified.

The questions were formulated based on the interview guide (Additional file 1: Fig. S1). The responses were then transcribed and transferred to NVivo (QSR International, Inc., Burlington, MA, USA), where thematic analysis was performed. Data were examined line by line, and the main codes and themes, felt to represent important concepts in answering the research questions, were identified using inductive analysis methods. Transcription and coding were completed by OAH and AG.

When differences existed, discussions took place between the team members to reach conclusions. Confidentiality was maintained, and all identifying information was removed. Each assessor had a unique reference code to track responses during the analysis phase. The authors followed the Consolidated Criteria for Reporting Qualitative Research (COREQ) checklist.

## Results

### Quantitative

Five examiners assessed the videos of each of the four residents. A total of 20 assessments were performed. Table 1 shows the demographic data of the examiners, and they have different scopes of practice including trauma, arthroplasty, sports, upper extremity, and spine surgery. Their years of experience range between 4 and 34 years with a mean of 18.4 years. The inter-rater reliability for video-based assessment among all examiners was 0.832, with a mean of 3.45 and a *p* value of 0.014 (Table 2).

**Table 1:** Specialty and years of experience of the examiners participated in VBA

Examiner	Specialty	Years of experience
1	Community arthroplasty and sports	4 years
2	Arthroplasty and trauma	34 years
3	Spine	20 years
4	Arthroplasty	17 years
5	Upper extremity and trauma	17 years

**Table 2:** Inter-rater reliability for video-based assessment

	IRR	Mean	CI	*p* value
Inter-rater reliability	0.832	3.45	0.247–0.988	0.014

### Qualitative

Major themes that were found in the interviews included the benefits and limitations of VBA (Table 3).

**Table 3:** Qualitative strengths and limitations of video-based assessment

Strengths	Limitations
Assesses global performance and/or specific skills at the same time	Poor audio–video quality
Potential use for uncommon procedures	
Ability to re-check missed points during live assessment	Inability to evaluate readiness for practice
More constructive feedback	Feedback could potentially be more time-consuming
Flexible evaluation time	
Can be done anonymously	
Can use multiple assessors	
Good teaching and coaching tool	

### Benefits

One of the major benefits of doing VBA is its ability to assess global performance. For instance, Interviewer 4 observed:

I thought with reviewing the video performance of the residents you can make generalized assessments of performance. I felt like you can see how the residents were working through procedures. You might miss little nuances about the plating technique, but you could see just how they get through that operation. You can see their ability to do it and you get a global assessment of what's happening. You can actually see that they actually accomplished the ability to get an exposure done in a certain time and get the plate on the bone with appropriate number of screws.

This idea was similarly summarized by Interviewer 5, who stated: “It does give you a nice sort of 360 evaluation of that person's thought process and abilities.”

Furthermore, it could also be used to assess specific skill sets and uncommon procedures that residents might not get enough exposure to during their training. Interviewer 5 stated: “I think you could use the videos to sign off on a certain skill set, to say yes they are capable of this operation,” while Interviewer 4 mentioned:

Where it could be helpful is using it for very particular procedures that are challenging for residents to get exposure to. Cases where residents need help to get the performance improved in a particular procedure that they're having trouble with.

Video-based assessment will allow the examiners to review and check on specific milestones while performing the assessment. We can also zoom in and out while performing VBA to look closely to minimize missing important checkpoints. This idea was reported by Interviewer 1, who explained:

it will remove, in theory, some of the things that we miss if we have attention lapses…. And still being able to review the process a few times, you would catch more deficiencies or more positives than in just watching the event in real time.

Similarly, Interviewer 4 highlighted that:

one good thing about it is [that] you can't reverse live. If you miss it live, you miss it completely. You take a little holiday with your focus, if you miss it you can't go back, whereas with the video, I was able to revert back and relook at things: did I see that? Is that what happened? I go back and look, [and] yeah, that actually did happen.

Video assessment can allow for more constructive and detailed feedback by using the videos to support learning from the evaluation. For instance, Interviewer 4 commented:

I think using the video to support that feedback will be powerful. I think it's better than just saying yeah, we looked at your video and thought you could have done this better and that better without them actually being able to see their performance where they're making mistakes.

Interviewer 5 also supported this idea, stating:

The nice thing about the simulation videos is that you have probably more consistent documented record of someone’s ability. I think it's constructive from the trainees’ perspective and that they can watch their performance afterwards and say could have done this better or that better or I wasn't very good at using that clamp.

Additionally, steps could be flagged, and comments could be added to the videos to be used during the feedback process. This point was illustrated by Interviewer 4:

I can imagine how it would be neat. You are an evaluator watching a video and you can put stop and flag. You can flag when something was being done. Here the resident is obviously not using the scissors right in dissecting in the correct plane. Then the resident when they're reviewing their performance could actually see points where their performance was being evaluated with actual comments written somewhere around it.

Videos can also be edited and used as a teaching tool for the future. Interviewer 2 expressed that “you could actually edit them and have them for teaching purposes, but actually now you don't know if that’s me doing it or the residents, which will be great.”

Another key benefit of VBA is the flexibility of evaluation time. It does not need to be done at the same time as the examination, and it can even be done outside normal work hours. Moreover, the examiners do not have to be present at the time of the actual test performance. This view was summarized by Interviewer 3: “I think the strength is the fact that you can uncouple the examiner from the day or the hour that the exam is taking place.” Likewise, Interviewer 4 described: “Looking at the videos is much more efficient from that standpoint, because it's just my computer, so I can sit there and look at it. So, from that standpoint, it's quicker and you can control what you're seeing.”

An additional advantage is that we can minimize bias by adding multiple assessors, making the assessment anonymous, or even blinding the examiners. They do not have to be physically attending the assessment, as it can be done at different training centers. Interviewer 1 illustrated that “having the ability to have multiple assessors, it could be possible to make it anonymous in a training program potentially.” Interviewer 2 added: “if you could mix it up and say they are from different schools; I think that way you take away the personal situation.” Similarly, Interviewer 3 expressed that:

video assessment will also allow us to have assessors from remote locations like have our residents examined by surgeons from other programs and vice versa. I also think that it allows for anonymization of the person which eliminates a problem particularly leads to bias.

### Limitations

The main challenge identified by the examiners was poor video and audio quality. A single audio input was used, which created background noise which was difficult for examiners to hear when trainees were explaining important steps of the procedure. It was difficult to know how much prompting was used. This important limitation was described by all participants. Interviewer 5 explained that the.

audio quality would have to be better both on the examiner’s part and on the examinee’s part so that you could clearly hear what questions are being asked and what the responses to those questions were and get a sense of how much prompting was done to arrive at the correct answer.

This was also expressed by Interviewer 1: “It was intermittently challenging because of the issues with a direct line of view and sometimes audio, so I had to go back and forth a lot of times to be able to get the evaluations.” Moreover, Interviewer 2 described that “I think the biggest problem was audio. It was sometimes noisy… just I felt I missed some of the answers and I just couldn't tell what exactly they were saying.” Interviewer 3’s comment was:

a single camera view that I don't think was adjusted often enough and the fact that it due to the audio you missed some of the very key components of the operation because it was apparent that the candidates were distracted to think out loud, but we weren’t able to assess it”.

Interviewer 4 does not think it is a fair evaluation method because of the video and audio quality:

I think from an evaluation standpoint a formal evaluation might not be as ideal because sometimes residents can be in and out of the video you might miss certain things that you can't see or hear. If this evaluation really matters like you need to hear or see certain aspects of the technical procedure being performed and you miss it and then that can negatively affect the evaluation.

While using a single camera view can provide an adequate view at the surgical field, it cannot show the whole interaction, which then inhibits a complete evaluation. Examiners described difficulty evaluating body language, nonverbal communication, and interaction with the assistant. Interviewer 4 described that “you couldn't actually see what the residents were doing with their body language and positioning. You might be missing out on some of the other stuff that's happening, such as how they are interacting with the assistant, for example.” Furthermore, Interviewer 5 added: “If a video is high enough fidelity… you would have better audio and multiple camera angles, both in the wound and then outside of the window, to see what they're doing and how they're directing their assistants.”

Additionally, we cannot yet use VBA to evaluate readiness for practice. Most examiners did not feel comfortable assessing readiness for practice from just watching those videos. For example, Interviewer 4 stated: “I don't think I would be comfortable evaluating readiness for professional practice based on video assessment just yet. At least the kind of video assessment that we had done.”

Lastly, Interviewer 5 was concerned that feedback could potentially be impractical and more time-consuming if we use the videos to give feedback to demonstrate important points:

If I went through it with the resident, I'm sure that for the training I would be pausing it to offer constructive feedback at various points to the video, to say like, ‘hey, you're not holding down the clamp right’, or ‘why did you cut away from your body instead of towards your body’. So, it would probably take longer if you reviewed the video with the resident.

## Discussion

To our knowledge, this is the first study to illustrate a good inter-rater reliability in using video-based assessment of open procedures in orthopedics (ICC = 0.832 and *p* value = 0.014). Few studies have yet demonstrated the reliability of VBA in other surgical specialties. Kulsoom et al. [17] evaluated the reliability of VBA of endoscopic sinus surgery performed by residents at different stages of their training on cadavers. Strong inter-rater reliability was shown. Similarly, Driscoll et al. studied the reliability of video assessment in comparison with real-time assessment of soft tissue handling while performing open inguinal hernia repair. They found that video assessment has equal ICC to real-time assessment and demonstrated a significant difference in scores in different years of training as well as consultants from the trainee level [18]. Birkmeyer et al. [19] most importantly demonstrated that surgical skill is directly correlated with patient outcomes in actual clinical scenarios making some form of objective assessment of open surgery imperative for training programs. In the orthopedic literature, VBA is reported as a reliable assessment method only for arthroscopic surgeries. For instance, Alvand et al. [20] validated a global rating scale to help assess residents’ learning curves in knee arthroscopic meniscal repair based on video assessment.

The qualitative part of this study adds to the strength of having a good inter-rater reliability and offers a description to help critically analyze the strengths and limitations of video-based assessment for future studies. Generally, examiners in this study had a positive impression of video-based assessment. Implementing VBA is believed to reduce the burden of direct live observational assessment and will therefore allow surgeons to concentrate more on patient outcomes and work efficiency. Likewise, allowing surgeons to concentrate on evaluation separately in the allocated time will consequently provide opportunity for a more comprehensive feedback process with high fidelity simulation [18].

This thematic analysis also demonstrated that VBA is a helpful tool for formative assessment, as it can gauge global performance and monitor progress. It can also be used for specific tasks or uncommon procedures and lead to more constructive feedback. On the other hand, most examiners were not comfortable using VBA for high-stakes summative evaluation or to assess readiness for practice. This is in agreement with a systematic review, which illustrated that while VBA can differentiate between assessment of examinees with a significant difference in their level of performance, it failed to assess small differences. Therefore, it was recommended that VBA only be used for formative assessment [21].

Examiners agreed that using the assessment videos for feedback and teaching will help improve residents’ surgical performance. Various forms of feedback on videos have been verified to improve performance. In a randomized controlled trial, Soucisse et al. compared 14 residents who had surgical video coaching to a control group of 14 other residents who had no coaching after performing side-to-side small bowel anastomosis. They showed a greater difference in Objective Structured Assessment of Technical (OSAT) skills score when they were asked to perform the same task again [22]. Naik et al. [23] demonstrated that suturing skills of interns can improve significantly after personalized audio feedback on their videos performing subcuticular wound closure. More interestingly, Aljamal et al. [24] showed that group feedback of videos of interns performing basic surgical skills is an effective method of feedback to improve performance.

Additionally, examiners elaborated on how this form of evaluation can be more convenient for them, for the following reasons: They do not have to be present during the actual examination, they can do the assessment at a convenient time, and they have control over the videos. These factors will theoretically improve the quality of the evaluations and feedback process as well as reduce examiner burnout. Moreover, distant evaluation allows the utilization of multiple examiners, which is believed to reduce bias and improve the quality of feedback [25]. This is also in agreement with another simulation-based video assessment study of 29 general surgery residents filmed performing two procedures (Laparoscopic Low Anterior Resection and Nissen Fundoplication). They were rated by ten assessors, with good inter-rater reliability of 0.74. In addition, they were able to reduce assessment time by 80% [26].

This pilot study has four main limitations. The first is the small sample size, as only four residents participated in the VBA. Having multiple assessors, however, helped to increase the sample size fivefold. This was similar to other studies in the surgical field where reliability assessment was performed using multiple expert assessors of a limited sample size [7, 27, 28]. The second limitation is that in this study, video-based assessments were performed for one procedure: open reduction and internal fixation of both bone forearm fracture. The results from this study cannot be generalized to all open orthopedic procedures as a single camera view may not be feasible to capture the surgical aspects of more complicated procedures like total hip replacement or more complex trauma surgeries. The third important limitation is the poor audio–video quality which continues to be a significant challenge in performing VBA. Lastly, the residents participating in the study were not interviewed about their feedback on VBA. That is, residents may have altered their behavior due to being video recorded, whether due to increased stress from the recording, or distraction due to the presence of the camera. However, each of these limitations could be addressed in future, larger studies, to provide a higher quality product for assessment. Future studies could include two simultaneous camera views for observers to evaluate, and surgeons and their assistants could wear small microphones to ensure that their communication is captured effectively. Additionally, surgical trainees could be interviewed in future studies to elicit their opinions on the use of VBA to evaluate their surgical skills.

## Conclusion

Video-based assessment for open orthopedic procedures is a potential reliable adjunct to live assessment, allowing for multiple examiners for the same procedure and more specific feedback for trainees. Despite good inter-rater reliability, surgeon assessors did not feel comfortable making decisions about progress or readiness for practice using the videos. Video and audio quality must be improved in future study before higher stakes decisions about progress can be evaluated.

## Supplementary Information

**Additional file 1. **Interview guide for semi-structured interviews with faculty staff doing video-based assessment.

## Data Availability

The datasets used and/or analyzed during the current study are available from the corresponding author on reasonable request.

## Abbreviations

CBME: Competency-Based Medical Education
VBA: Video-based assessment
S-OSCE: Surgical Objective Structured Clinical Examination
ORIF: Open reduction internal fixation
RCPSC: Royal College of Physicians and Surgeons of Canada
O-SCORE: Ottawa Surgical Competency Operating Room Evaluation
ICC: Intraclass correlation coefficient
