# Supplementary material for: Video-based assessment (VBA) of an open, simulated orthopedic surgical procedure: a pilot study using a single-angle camera to assess surgical skill and decision making
Source: J Orthop Surg Res. 2023 Feb 7;18:90. doi: 10.1186/s13018-023-03557-3 (PMC9904250; doi:10.1186/s13018-023-03557-3)
Supplement: Supplementary file 1 — Additional file 1. Interview guide for semi-structured interviews with faculty staff doing video-based assessment. [file 13018_2023_3557_MOESM1_ESM.docx]

**Supplemental Material:** Interview guide for semi-structured interviews with faculty staff doing video-based assessment

1. Please share your experience in medical training and what role simulation played in this training.
2. Please describe your experience in the evaluation of technical skills of residents:
   1. Have you attended courses on proper evaluation techniques for surgical skills?
   2. Have you had any formal training on how to assess surgical skills in the lab? Please elaborate on whether you think faculty development is needed.
3. What value do you think surgical videos brings to teaching and therefore potentially to assessing surgical skills?
4. How would you describe your experience doing this video-based assessment?
5. How would you describe the quality of these videos?
6. What do you think are the strengths of video-based assessment?
   - - 1. Do you think you can evaluate for readiness for practice? Please elaborate.
       2. Do you think you can differentiate between PGY3 and PGY5 residents?
   1. What limitations exist for this evaluation tool?
      - 1. Elaborate on the time this evaluation took you.
        2. Is this feasible for delivering appropriate and timely feedback for residents?
7. Do you think it is a fair way to replace the current resident evaluation in the operating room?
8. What would you suggest for improving this experience?
9. Would you do it again? Please elaborate why or why not.
